# A mathematical model to guide the re-opening of economies during the COVID-19 pandemic

**DOI:** 10.1016/j.amsu.2020.06.041

**Published:** 2020-07-11

**Authors:** Habib Noorbhai

**Affiliations:** Faculty of Health Sciences, University of Johannesburg, Doornfontein Campus, Office 5306C, 5th floor, John Orr Building, Johannesburg, South Africa

**Keywords:** Coronavirus, Lockdown, Model, Norms, Health, Economy

## Abstract

Despite rigorous global containment and quarantine efforts, the incidence of the Severe Acute Respiratory Syndrome Coronavirus 2 (SARS-CoV-2), also known as COVID-19, continues to surge, with more than 12 million laboratory-confirmed cases and over 500,000 deaths worldwide (as of 11 July 2020). Aside from the continued surge in cases and the imperatives of public health concern and saving lives, economic devastation is also mounting with a global depression now seeming inevitable. There is limited attention directed towards people who have recovered from the virus and whether this metric can be useful in guiding when the economy can be re-opened. In this paper, a simpler model is presented in order to guide various countries on the (possible) re-opening of the economy (or re-opening in stages/phases) alongside risk categories and ratios. Factors that need to be considered when applying the model include the healthcare capacity in terms of the number of hospitals, beds and healthcare workers that are available to capacitate this virus. In addition, population size, physical distancing measures, socio-economic disparities, lockdown regulations in each country, and more importantly - the amount and accuracy of testing conducted, is also imperative to consider. Decisions adopted by leaders around the world have the most difficult decision to make (yet), and have to weigh up on what really matters; health or wealth. It is suggested that this model be applied in a number of states/counties and countries in order to gauge the risk of their location being re-opened, by observing their total number of recoveries in proximity to total number of cases.

Despite rigorous global containment and quarantine efforts, the incidence of the Severe Acute Respiratory Syndrome Coronavirus 2 (SARS-CoV-2), also known as COVID-19, continues to surge, with more than 12 million laboratory-confirmed cases and over 500,000 deaths worldwide (as of July 11, 2020) [1]. While the trajectory of this outbreak is impossible to predict, effective response requires prompt action from the standpoint of classic public health strategies to the timely development and implementation of effective countermeasures [2].

Aside from the continued surge in cases and the imperatives of public health concern and saving lives, economic devastation is also mounting with a global depression now seeming inevitable. It is estimated that the gross domestic product (GDP) growth would be massively affected, ranging from 3 to 6% (depending on the country) [3]. As a consequence, in approximately 30 countries, a median decline in GDP in 2020 of −2.8% would be observed. In other scenarios, the GDP can fall more than 10%, and in some countries, more than 15% [3]. It is further estimated that on average, each additional month of lockdown or restrictions will cost 2.5–3% of global GDP. It is projected that if the ongoing crisis lasts beyond July 2020, the global economy faces the gravest threat seen in the last two centuries [3].

Based on the above, there has been an overwhelming amount of attention dedicated towards the number of cases and deaths per day, globally, and within each country. However, there is limited attention directed towards people who have recovered from the virus and whether this metric can be useful in guiding when the economy can be re-opened. It has been suggested that mathematical modelling is a powerful tool for understanding transmission of COVID-19 and exploring different scenarios [4]. One model looked at different policies that yield the same transmission rate and have the same health outcomes but can have very different economic costs [5]. It has also been highlighted that understanding the dynamics of case-fatality and recovery rates of COVID-19 would enhance the knowledge base on the current trends of the severity of the epidemic [6]. However, there are no papers documenting models in conjunction with risk categories and correlating these to specific ratio values. In this paper, a simpler model is presented in order to guide various countries on the (possible) re-opening of the economy (or re-opening in stages/phases) alongside risk categories and ratios.

Total Recoveries = RT | Total Cases = CT | Ratio = r

Mathematical Model: RT / CT = r

Example: Globally, as of 11 July, there are approximately 6,740,000 recoveries and 12,300,000 cases.

This would work out as: 6,740,000/12,300,000 = a ratio of 0.55.

Below, Table 1 guides the risk-to-ratio guide for re-opening of the economies out of lockdown. Where r is 0.0–0.40, it is a high risk; 0.41–0.66 is a moderate risk; 0.67–0.85 is a mild risk; and 0.85–0.99 is a low risk. The risk category classifications (high, moderate, mild and low) are based on a number of countries who have opened their economy after seeing a reduction in the amount of COVID-19 cases. The risk category classifications are, however, generic as each state/county and country would need to use it as a guide in order to apply the ratio to their unique determinants, circumstances and situation, population size, as well as the level/phase of the restrictions. Following this guide would also allow leaders to reduce the complacency towards the virus and assist in the re-opening of their economy/country.

**Table 1:** Risk/ratio guide for re-opening of the economies out of lockdown.

Ratio	Risk	Level/Phase
0.0–0.40	High	5
0.41–0.66	Moderate	4
0.67–0.85	Mild	3
0.86–0.99	Low	2

The United States of America have a high risk ratio of 0.30, whereas Italy and Australia (with an imminent outbreak) have a mild risk ratio of 0.80 and 0.81, respectively. More concerningly, the United Kingdom has an extremely high risk ratio of 0.003 with a severely low number of recoveries (n = 780, out of approximately 288,000 infected cases).

Despite the above model and normative guide, caution must be exercised when applying it in each country. One theory that comes to mind is the social determinants of health inequalities. These determinants are important to consider and include: income and social status, social support networks, employment and working conditions, physical environments, education, healthy child development, biology and genetic endowment, health services, personal health practices (including hygiene) and coping skills, and transport [7].

An important factor that has recently been found is the vulnerability of the poor and the power of the privileged in a pandemic. This indicates that those most vulnerable will be worse affected [8]. It is also suggested that the health promotion community must ensure that considerations of health equity and social justice principles remain at the forefront of the pandemic responses [9]. This will not be so simple, especially at a time when varied stakeholders pitch population health against national economic stability [8].

Other factors include the healthcare capacity in terms of the number of hospitals, beds and healthcare workers that are available to capacitate this virus. In addition, population size, physical distancing measures, socio-economic disparities, lockdown regulations in each country, and more importantly - the amount and accuracy of testing conducted, is also imperative to consider [10].

Decisions adopted by leaders around the world have the most difficult decision to make (yet), and have to weigh up on what really matters; health or wealth. This mathematical model has not been trialled or validated against any other models to provide evidence towards its applicability across different countries and settings. It is, therefore, suggested that this model be applied in a number of states/counties and countries in order to gauge the risk of their location being re-opened, by observing their total number of recoveries in proximity to total number of cases.

## Sources of funding

None.

## Author contribution

HN conceptualised and wrote the paper.

## Consent

N/A.

## Registration of research studies

1. Name of the registry: N/A
2. Unique Identifying number or registration ID: N/A
3. Hyperlink to your specific registration (must be publicly accessible and will be checked): N/A

## Guarantor

HN.

## Provenance and peer review

Not commissioned, externally reviewed.

## Ethical approval

N/A.

## Declaration of competing interest

None.
